# Supplementary material for: Potential Role of PSMA-Targeted PET in Thyroid Malignant Disease: A Systematic Review
Source: Diagnostics (Basel). 2023 Feb 3;13(3):564. doi: 10.3390/diagnostics13030564 (PMC9914698; doi:10.3390/diagnostics13030564)
Supplement: Supplementary file 1 [file diagnostics-13-00564-s001.zip › Supplementary Table S2.pdf]

Table S2. list of excluded studies with reasons.

Excluded Studies	Reason for exclusion
Verburg, F.A.; Krohn, T.; Heinzel, A.; Mottaghy, F.M.; Behrendt, F.F. First Evidence of PSMA Expression in Differentiated Thyroid Cancer Using [^{68}Ga]PSMA-HBED-CC PET/CT. *Eur J Nucl Med Mol Imaging* **2015**, *42*, 1622–1623, doi:10.1007/s00259-015-3065-y.	Case report in the field of interest
Damle, N.A.; Tripathi, M.; Chakraborty, P.S.; Sahoo, M.K.; Bal, C.; Aggarwal, S.; Arora, G.; Kumar, P.; Kumar, R.; Gupta, R. Unusual Uptake of Prostate Specific Tracer 68Ga-PSMA-HBED-CC in a Benign Thyroid Nodule. *Nucl Med Mol Imaging* **2016**, *50*, 344–347, doi:10.1007/s13139-016-0408-y.	Case report in the field of interest
Demirci, E.; Sahin, O.E.; Ocak, M.; Akovali, B.; Nematyazar, J.; Kabasakal, L. Normal Distribution Pattern and Physiological Variants of 68Ga-PSMA-11 PET/CT Imaging. *Nucl Med Commun* **2016**, *37*, 1169–1179, doi:10.1097/MNM.0000000000000566.	Original study not in the field of interest
Gupta, M.; Choudhury, P.S.; Gupta, G.; Gandhi, J. Metastasis in Urothelial Carcinoma Mimicking Prostate Cancer Metastasis in Ga-68 Prostate-Specific Membrane Antigen Positron Emission Tomography-Computed Tomography in a Case of Synchronous Malignancy. *Indian J Nucl Med* **2016**, *31*, 222–224, doi:10.4103/0972-3919.183615.	Case report not in the field of interest
Kanthan, G.L.; Drummond, J.; Schembri, G.P.; Izard, M.A.; Hsiao, E. Follicular Thyroid Adenoma Showing Avid Uptake on 68Ga PSMA-HBED-CC PET/CT. *Clin Nucl Med* **2016**, *41*, 331–332, doi:10.1097/RLU.0000000000001084.	Case report in the field of interest
Shetty, D.; Loh, H.; Bui, C.; Mansberg, R.; Stevanovic, A. Elevated 68Ga Prostate-Specific Membrane Antigen Activity in Metastatic Non-Small Cell Lung Cancer. *Clin Nucl Med* **2016**, *41*, 414–416, doi:10.1097/RLU.0000000000001139.	Case report not in the field of interest
Langsteger, W.; Rezaee, A.; Loidl, W.; Geinitz, H.S.; Fitz, F.; Steinmair, M.; Broinger, G.; Pallwien-Prettner, L.; Beheshti, M.; Imamovic, L.; et al. 32nd International Austrian Winter Symposium : Zell Am See, the Netherlands. 20-23 January 2016. *EJNMMI Res* **2016**, *6*, 32, doi:10.1186/s13550-016-0168-9.	Collection of symposium abstracts
Sager, S.; Vatankulu, B.; Uslu, L.; Sönmezoglu, K. Incidental Detection of Follicular Thyroid Carcinoma in 68Ga-PSMA PET/CT Imaging. *J Nucl Med Technol* **2016**, *44*, 199–200, doi:10.2967/jnmt.115.171660.	Case report in the field of interest
Sasikumar, A.; Joy, A.; Nanabala, R.; Unni, M.; Tk, P. Complimentary Pattern of Uptake in 18F-FDG PET/CT and 68Ga-Prostate-Specific Membrane Antigen PET/CT in a Case of Metastatic Clear Cell Renal Carcinoma. *Clin Nucl Med* **2016**, *41*, e517–e519, doi:10.1097/RLU.0000000000001394.	Case report not in the field of interest
Taywade, S.K.; Damle, N.A.; Bal, C. PSMA Expression in Papillary Thyroid Carcinoma: Opening a New Horizon in Management of Thyroid Cancer? *Clin Nucl Med* **2016**, *41*, e263-265, doi:10.1097/RLU.0000000000001148.	Case report in the field of interest
Choudhury, P.; Gupta, M. Personalized & Precision Medicine in Cancer: A Theranostic Approach. *Curr Radiopharm* **2017**, *10*, 166–170, doi:10.2174/1874471010666170728094008.	Review not in the field of interest
Derlin, T.; Kreipe, H.-H.; Schumacher, U.; Soudah, B. PSMA Expression in Tumor Neovasculature Endothelial Cells of Follicular Thyroid Adenoma as Identified by Molecular Imaging Using 68Ga-PSMA Ligand PET/CT. *Clin Nucl Med* **2017**, *42*, e173–e174, doi:10.1097/RLU.0000000000001487.	Case report in the field of interest

Jena, A.; Zaidi, S.; Kashyap, V.; Jha, A.; Taneja, S. PSMA Expression in Multinodular Thyroid Neoplasm on Simultaneous Ga-68-PSMA PET/MRI. *Indian J Nucl Med* **2017**, *32*, 159–161, doi:10.4103/0972-3919.202248.	Case report in the field of interest
Kirchner, J.; Schaarschmidt, B.M.; Sawicki, L.M.; Heusch, P.; Hautzel, H.; Ermert, J.; Rabenalt, R.; Antoch, G.; Buchbender, C. Evaluation of Practical Interpretation Hurdles in 68Ga-PSMA PET/CT in 55 Patients: Physiological Tracer Distribution and Incidental Tracer Uptake. *Clin Nucl Med* **2017**, *42*, e322–e327, doi:10.1097/RLU.0000000000001672.	Case report in the field of interest
Luster, M.; Pfestroff, A.; Verburg, F.A. Recent Advances in Nuclear Medicine in Endocrine Oncology. *Curr Opin Oncol* **2017**, *29*, 1–6, doi:10.1097/CCO.0000000000000338.	Review in the field of interest
Osman, M.M.; Iravani, A.; Hicks, R.J.; Hofman, M.S. Detection of Synchronous Primary Malignancies with 68Ga-Labeled Prostate-Specific Membrane Antigen PET/CT in Patients with Prostate Cancer: Frequency in 764 Patients. *J Nucl Med* **2017**, *58*, 1938–1942, doi:10.2967/jnumed.117.190215.	Original study not in the field of interest
Sjögreen Gleisner, K.; Spezi, E.; Solny, P.; Gabina, P.M.; Cicone, F.; Stokke, C.; Chiesa, C.; Paphiti, M.; Brans, B.; Sandström, M.; et al. Variations in the Practice of Molecular Radiotherapy and Implementation of Dosimetry: Results from a European Survey. *EJNMMI Phys* **2017**, *4*, 28, doi:10.1186/s40658-017-0193-4.	Original study not in the field of interest
Zacho, H.D.; Nielsen, J.B.; Dettmann, K.; Haberkorn, U.; Petersen, L.J. Incidental Detection of Thyroid Metastases From Renal Cell Carcinoma Using 68Ga-PSMA PET/CT to Assess Prostate Cancer Recurrence. *Clin Nucl Med* **2017**, *42*, 221–222, doi:10.1097/RLU.0000000000001522.	Case report not in the field of interest
Arora, S.; Damle, N.A.; Parida, G.K.; Singhal, A.; Nalli, H.; Dattagupta, S.; Bal, C. Recurrent Medullary Thyroid Carcinoma on 68Ga-Prostate-Specific Membrane Antigen PET/CT: Exploring New Theranostic Avenues. *Clin Nucl Med* **2018**, *43*, 359–360, doi:10.1097/RLU.0000000000002010.	Case report not in the field of interest
Damle, N.A.; Bal, C.; Singh, T.P.; Gupta, R.; Reddy, S.; Kumar, R.; Tripathi, M. Anaplastic Thyroid Carcinoma on 68 Ga-PSMA PET/CT: Opening New Frontiers. *Eur J Nucl Med Mol Imaging* **2018**, *45*, 667–668, doi:10.1007/s00259-017-3904-0	Case report in the field of interest
Keidar, Z.; Gill, R.; Goshen, E.; Israel, O.; Davidson, T.; Morgulis, M.; Pirmisashvili, N.; Ben-Haim, S. 68Ga-PSMA PET/CT in Prostate Cancer Patients - Patterns of Disease, Benign Findings and Pitfalls. *Cancer Imaging* **2018**, *18*, 39, doi:10.1186/s40644-018-0175-3.	Original study not in the field of interest
Malik, D.; Sood, A.; Mittal, B.R.; Singh, H.; Basher, R.K.; Shukla, J.; Bhattacharya, A.; Singh, S.K. Nonspecific Uptake of 68Ga-Prostate-Specific Membrane Antigen in Diseases Other than Prostate Malignancy on Positron Emission Tomography/Computed Tomography Imaging: A Pictorial Assay and Review of Literature. *Indian J Nucl Med* **2018**, *33*, 317–325, doi:10.4103/ijnm.IJNM_81_18.	Review not in the field of interest
Sasikumar, A.; Joy, A.; Pillai, M.R.A.; Oommen, K.E.; Jayakumar, R. Rare Case of Intratracheal Metastasis Detected on 68Ga-Prostate-Specific Membrane Antigen PET/CT Scan in a Case of Thyroglobulin Elevated Negative Iodine Scan Syndrome. *Clin Nucl Med* **2018**, *43*, 282–283, doi:10.1097/RLU.0000000000001992.	Case report in the field of interest
Singh, D.; Horneman, R.; Nagra, N.K. More than the Prostate: Intrapancreatic Accessory Spleen and Papillary Thyroid Cancer Detected with 18F-PSMA PET/CT. *Hell J Nucl Med* **2018**, *21*, 145–147, doi:10.1967/s002449910805.	Case report in the field of interest

Bertagna, F.; Albano, D.; Giovanella, L.; Bonacina, M.; Durmo, R.; Giubbini, R.; Treglia, G. 68Ga-PSMA PET Thyroid Incidentalomas. *Hormones (Athens)* **2019**, *18*, 145–149, doi:10.1007/s42000-019-00106-8.	Review in the field of interest
Ciappuccini, R.; Edet-Sanson, A.; Saguet-Rysanek, V.; Gauthé, M.; Bardet, S. Thyroid Incidentaloma on 18F-Fluorocholine PET/CT and 68Ga-PSMA PET/CT Revealing a Medullary Thyroid Carcinoma. *Clin Nucl Med* **2019**, *44*, 663–665, doi:10.1097/RLU.0000000000002559.	Case report in the field of interest
Langbein, T.; Weber, W.A.; Eiber, M. Future of Theranostics: An Outlook on Precision Oncology in Nuclear Medicine. *J Nucl Med* **2019**, *60*, 13S-19S, doi:10.2967/jnumed.118.220566.	Review not in the field of interest
Lengana, T.; Lawal, I.O.; Mokoala, K.; Vorster, M.; Sathekge, M.M. 68Ga-PSMA: A One-Stop Shop in Radioactive Iodine Refractory Thyroid Cancer? *Nucl Med Mol Imaging* **2019**, *53*, 442–445, doi:10.1007/s13139-019-00621-x.	Review not in the field of interest
Mai, T.K.; Pham, C.P.; Bui, T.C.; Tran, H.B.; Pham, V.T.; Dao, M.P.; Tran, D.H. Efforts in the Formation and Development of Nuclear Medicine in Vietnam. *Nucl Med Mol Imaging* **2019**, *53*, 83–85, doi:10.1007/s13139-018-00570-x.	Review not in the field of interest
Ngoc, C.N.; Happel, C.; Sabet, A.; Bechstein, W.O.; Grünwald, F. Iodine Avid Papillary Thyroid Cancer Showing PSMA-Expression in 68Ga-PSMA Ligand PET/CT. *Nuklearmedizin* **2019**, *58*, 50–51, doi:10.1055/a-0808-0832.	Case report in the field of interest
Annovazzi, A.; Faiella, A.; Pescarmona, E.; Sanguineti, G.; Sciuto, R. Asymptomatic Metastasis to Thyroid Cartilage Detected by 18F-Choline and 64Cu-PSMA PET/CT as a Single Site of Disease Relapse in a Patient With Castration-Resistant Prostate Carcinoma. *Clin Nucl Med* **2020**, *45*, 214–216, doi:10.1097/RLU.0000000000002786.	Case report not in the field of interest
Chen, W.; Lee, Z.; Awadallah, A.; Zhou, L.; Xin, W. Peritumoral/Vascular Expression of PSMA as a Diagnostic Marker in Hepatic Lesions. *Diagn Pathol* **2020**, *15*, 92, doi:10.1186/s13000-020-00982-4.	Original study not in the field of interest
Filippi, L.; Chiaravalloti, A.; Schillaci, O.; Cianni, R.; Bagni, O. Theranostic Approaches in Nuclear Medicine: Current Status and Future Prospects. *Expert Rev Med Devices* **2020**, *17*, 331–343, doi:10.1080/17434440.2020.1741348.	Review not in the field of interest
Filippov, A.; Bonjoc, K.-J.C.; Chea, J.; Bowles, N.; Poku, E.; Chaudhry, A. Role of Theranostics in Thoracic Oncology. *J Thorac Dis* **2020**, *12*, 5140–5146, doi:10.21037/jtd-2019-pitd-13.	Review not in the field of interest
Gossili, F.; Petersen, L.J.; Zacho, H.D. The Frequency of Thyroid Incidental Findings and Risk of Malignancy Detected by 68Ga-Labeled Prostate-Specific Membrane Antigen PET/CT in Prostate Cancer. *Hell J Nucl Med* **2020**, *23*, 240–245, doi:10.1967/s002449912202.	Case report in the field of interest
Mohan, V.; Vogel, W.V.; Valk, G.D.; de Boer, J.P.; Lam, M.G.E.H.; de Keizer, B. PSMA PET/CT Identifies Intrapatient Variation in Salivary Gland Toxicity From Iodine-131 Therapy. *Mol Imaging* **2020**, *19*, 1536012120934992, doi:10.1177/1536012120934992.	Original study not in the field of interest
Santhanam, P.; Russell, J.; Rooper, L.M.; Ladenson, P.W.; Pomper, M.G.; Rowe, S.P. The Prostate-Specific Membrane Antigen (PSMA)-Targeted Radiotracer 18F-DCFPyL Detects Tumor Neovasculature in Metastatic, Advanced, Radioiodine-Refractory, Differentiated Thyroid Cancer. *Med Oncol* **2020**, *37*, 98, doi:10.1007/s12032-020-01427-0.	Small case series in the field of interest
Sood, A.; Vadi, S.K.; Kumar, R.; Singh, H.; Mittal, B.R. Incidental Detection of Hypothyroidism on 68Ga-PSMA-HBED-CC PET/CT Imaging. *Clin Nucl Med* **2020**, *45*, e217–e218, doi:10.1097/RLU.0000000000002965.	Case report in the field of interest
Tang, K.; Wang, Z.; Lin, J.; Zheng, X. Hürthle Cell Thyroid Adenoma	Case report in the field of

Showing Avid Uptake on 18F-PSMA-1007 PET/CT. *Clin Nucl Med* **2020**, *45*, 223–224, doi:10.1097/RLU.0000000000002873.	interest
Tupalli, A.; Damle, N.A.; Thankarajan, A.S.; Mangu, B.S.; Kumar, A.; Khan, D.; Sagar, S.; Bal, C. An Unusual Case of Simultaneous Cricoid and Thyroid Cartilage Metastases from Prostatic Adenocarcinoma on 68Ga-PSMA PET/CT. *Nucl Med Mol Imaging* **2020**, *54*, 61–62, doi:10.1007/s13139-019-00625-7.	Case report in the field of interest
Aksoy, O.; Pencik, J.; Hartenbach, M.; Moazzami, A.A.; Schlederer, M.; Balber, T.; Varady, A.; Philippe, C.; Baltzer, P.A.; Mazumder, B.; et al. Thyroid and Androgen Receptor Signaling Are Antagonized by μ-Crystallin in Prostate Cancer. *Int J Cancer* **2021**, *148*, 731–747, doi:10.1002/ijc.33332	Original study not in the field of interest
Ciappuccini, R.; Saguet-Rysanek, V.; Giffard, F.; Licaj, I.; Dorbeau, M.; Clarisse, B.; Poulain, L.; Bardet, S. PSMA Expression in Differentiated Thyroid Cancer: Association With Radioiodine, 18FDG Uptake, and Patient Outcome. *J Clin Endocrinol Metab* **2021**, *106*, 3536–3545, doi:10.1210/clinem/dgab563.	Case report in the field of interest
Civan, C.; Isik, E.G.; Simsek, D.H. Metastatic Poorly Differentiated Thyroid Cancer With Heterogeneous Distribution of 18F-FDG, 68Ga-DOTATATE, and 68Ga-PSMA on PET/CT. *Clin Nucl Med* **2021**, *46*, e212–e213, doi:10.1097/RLU.0000000000003362.	Case report in the field of interest
Freudenberg, L.S.; Essler, M.; Herrmann, K. [Impact of COVID-19 on Nuclear Medicine Procedures in Germany 2020 - Results of a National Survey]. *Nuklearmedizin* **2021**, *60*, 210–215, doi:10.1055/a-1446-7641.	Survey not in the field of interest
Klain, M.; Zampella, E.; Nappi, C.; Nicolai, E.; Ambrosio, R.; Califaretti, E.; Lamartina, L.; Schlumberger, M.; Deandreis, D.; Salvatore, D.; et al. Advances in Functional Imaging of Differentiated Thyroid Cancer. *Cancers (Basel)* **2021**, *13*, 4748, doi:10.3390/cancers13194748.	Review not in the field of interest
Li, H.; Liu, Y.; Zang, J.; Yang, Z.; Lin, Y. Inferior Vena Cava Tumor Thrombus From Thyroid Cancer Detected by 68Ga-PSMA-617 PET/CT. *Clin Nucl Med* **2021**, *46*, 264–265, doi:10.1097/RLU.0000000000003497.	Case report in the field of interest
Mohan, V.; Bruin, N.M.; Tesselaar, M.E.T.; de Boer, J.P.; Vegt, E.; Hendrikx, J.J.M.A.; Al-Mamgani, A.; van de Kamer, J.B.; Sonke, J.-J.; Vogel, W.V. Muscarinic Inhibition of Salivary Glands with Glycopyrronium Bromide Does Not Reduce the Uptake of PSMA-Ligands or Radioiodine. *EJNMMI Res* **2021**, *11*, 25, doi:10.1186/s13550-021-00770-1.	Original study not in the field of interest
Sonavane, S.N.; Basu, S. Differential Tumor Biology between Locoregional and Distant Metastasis in a Patient with TENIS with TKI-Resistant Aggressive Recurrent Disease: A Comparative Evaluation with FDG, 68Ga-DOTATATE and 68Ga-PSMA-11 PET-CT. *J Nucl Med Technol* **2021**, jnmt.121.263452, doi:10.2967/jnmt.121.263452.	Case report in the field of interest
Uijen, M.J.M.; Derks, Y.H.W.; Merkx, R.I.J.; Schilham, M.G.M.; Roosen, J.; Privé, B.M.; van Lith, S. a. M.; van Herpen, C.M.L.; Gotthardt, M.; Heskamp, S.; et al. PSMA Radioligand Therapy for Solid Tumors Other than Prostate Cancer: Background, Opportunities, Challenges, and First Clinical Reports. *Eur J Nucl Med Mol Imaging* **2021**, *48*, 4350–4368, doi:10.1007/s00259-021-05433-w.	Review in the field of interest
Usmani, S.; Al-Turkait, D.; Al-Kandari, F.; Ahmed, N. Thyroid Cancer Detected on 68Ga-PMSA PET/CT. *J Pak Med Assoc* **2021**, *71*, 1511–1512.	Case report in the field of interest
Verma, P.; Malhotra, G.; Meshram, V.; Chandak, A.; Sonavane, S.; Lila, A.R.; Bandgar, T.R.; Asopa, R.V. Prostate-Specific Membrane Antigen Expression in Patients With Differentiated Thyroid Cancer With	Case report in the field of interest

Thyroglobulin Elevation and Negative Iodine Scintigraphy Using 68Ga-PSMA-HBED-CC PET/CT. *Clin Nucl Med* **2021**, *46*, e406–e409, doi:10.1097/RLU.0000000000003655.	
Bal, C.; Chakraborty, D.; Khan, D. Positron Emission Tomography/Computed Tomography in Thyroid Cancer. *PET Clin* **2022**, *17*, 265–283, doi:10.1016/j.cpet.2021.12.004.	Review in the field of interest
Califaretti, E.; Dall'armellina, S.; Rovera, G.; Finessi, M.; Deandreis, D. The Role of PET/CT in Thyroid Autoimmune Diseases. *Q J Nucl Med Mol Imaging* **2022**, *66*, 218–228, doi:10.23736/S1824-4785.22.03464-1.	Review not in the field of interest
Dittrich, R.P.; De Jesus, O. Gallium Scan. In *StatPearls*; StatPearls Publishing: Treasure Island (FL), 2022.	Book chapter not in the field of interest
Eilsberger, F.; Luster, M.; Librizzi, D.; Rodepeter, F.; Holzer, K.; Pfestroff, A. Medullary Thyroid Carcinoma Presenting as an Incidentaloma on Gallium-68-PSMA-PET/CT - Systematic Literature Review and Case Report. *Nuklearmedizin* **2022**, *61*, 458–461, doi:10.1055/a-1896-0106.	Case report and literature review in the field of interest
Fortunati, E.; Argalia, G.; Zanoni, L.; Fanti, S.; Ambrosini, V. New PET Radiotracers for the Imaging of Neuroendocrine Neoplasms. *Curr Treat Options Oncol* **2022**, *23*, 703–720, doi:10.1007/s11864-022-00967-z.	Review not in the field of interest
Freesmeyer, M.; Gühne, F.; Drescher, R.; Winkens, T.; Gassler, N.; Seifert, P. Multimodal Characterization of a PSMA-Positive Thyroid Nodule Using 68Ga-PSMA and 124Iodine PET/US Fusion Imaging. *Diagnostics (Basel)* **2022**, *12*, 472, doi:10.3390/diagnostics12020472.	Case report in the field of interest
Hasenauer, N.; Higuchi, T.; Deschler-Baier, B.; Hartrampf, P.E.; Pomper, M.G.; Rowe, S.P.; Fassnacht, M.; Buck, A.K.; Werner, R.A. Visualization of Tumor Heterogeneity in Advanced Medullary Thyroid Carcinoma by Dual-Tracer Molecular Imaging: Revealing the Theranostic Potential of SSTR- and PSMA-Directed Endoradiotherapy. *Clin Nucl Med* **2022**, *47*, 651–652, doi:10.1097/RLU.0000000000004082.	Case report in the field of interest
Leder, T.; Drescher, R.; Gühne, F.; Theis, B.; Freesmeyer, M. De Quervain Subacute Thyroiditis With Moderate PSMA Uptake Mimicking Thyroid Metastasis of Renal Cell Carcinoma. *Clin Nucl Med* **2022**, *47*, 221–222, doi:10.1097/RLU.0000000000003952.	Case report not in the field of interest
Lu, Y. Imaging Characteristics of Coexisting Metastatic Papillary Thyroid Cancer and Prostate Cancer on 18 F-Fluciclovine and 68 Ga-PSMA-11 PET/CT. *Clin Nucl Med* **2022**, *47*, 820–821, doi:10.1097/RLU.0000000000004156.	Case report in the field of interest
Parghane, R.V.; Basu, S. 18F-FDG PET/CT vs. 68Ga-PSMA-11 PET/CT in Evaluation of Distant Metastatic Disease in Recurrent Renal Cell Carcinoma. *J Nucl Med Technol* **2022**, jnmt.122.264014, doi:10.2967/jnmt.122.264014.	Case report not in the field of interest
Parghane, R.V.; Basu, S. Thyroid Incidentaloma on 68Ga-PSMA-11 PET/CT Leading to Detection of Thyroid Metastasis in Metastatic Prostate Carcinoma. *Clin Nucl Med* **2022**, *47*, e300–e301, doi:10.1097/RLU.0000000000003954.	Case report in the field of interest
Piek, M.W.; de Vries, L.H.; Donswijk, M.L.; de Keizer, B.; de Boer, J.P.; Lodewijk, L.; van Leeuwaarde, R.S.; Vriens, M.R.; Hartemink, K.J.; van der Ploeg, I.M.C. Retrospective Analysis of PSMA PET/CT Thyroid Incidental Uptake in Adults: Incidence, Diagnosis, and Treatment/Outcome in a Tertiary Cancer Referral Center and University Medical Center. *Eur J Nucl Med Mol Imaging* **2022**, *49*, 2392–2400, doi:10.1007/s00259-022-05679-y.	Original article not in the field of interest
Rizzo, A.; Dall'Armellina, S.; Pizzuto, D.A.; Perotti, G.; Zagaria, L.; Lanni, V.; Treglia, G.; Racca, M.; Annunziata, S. PSMA Radioligand Uptake as a	Review in the field of interest

Biomarker of Neoangiogenesis in Solid Tumours: Diagnostic or Theragnostic Factor? *Cancers (Basel)* **2022**, *14*, 4039, doi:10.3390/cancers14164039.	
Rosar, F.; Burgard, C.; Neubert, C.; Stahl, P.R.; Khreish, F.; Ezziddin, S. PSMA-Positive Follicular Thyroid Carcinoma Incidentally Detected by [68Ga]Ga-PSMA-11 PET/CT: Correlation with Immunohistology Confirms Neovascular PSMA-Expression. *Diagnostics (Basel)* **2022**, *12*, 1211, doi:10.3390/diagnostics12051211.	Case report in the field of interest
Piek, M.W.; de Vries, L.H.; Donswijk, M.L.; de Keizer, B.; de Boer, J.P.; Lodewijk, L.; van Leeuwaarde, R.S.; Vriens, M.R.; Hartemink, K.J.; van der Ploeg, I.M.C. Retrospective Analysis of PSMA PET/CT Thyroid Incidental Uptake in Adults: Incidence, Diagnosis, and Treatment/Outcome in a Tertiary Cancer Referral Center and University Medical Center. *Eur J Nucl Med Mol Imaging* **2022**, *49*, 2392–2400, doi:10.1007/s00259-022-05679-y.	Original article not in the field of interest
Schmidt, M.; Bartenstein, P.; Bucerius, J.; Dietlein, M.; Drzezga, A.; Herrmann, K.; Lapa, C.; Lorenz, K.; Musholt, T.J.; Nagarajah, J.; et al. Individualized Treatment of Differentiated Thyroid Cancer: The Value of Surgery in Combination with Radioiodine Imaging and Therapy - A German Position Paper from Surgery and Nuclear Medicine. *Nuklearmedizin* **2022**, *61*, 87–96, doi:10.1055/a-1783-8154.	Review not in the field of interest
Schmidt, M.; Bartenstein, P.; Bucerius, J.; Dietlein, M.; Drzezga, A.; Herrmann, K.; Lapa, C.; Lorenz, K.; Musholt, T.J.; Nagarajah, J.; et al. Correction: Individualized Treatment of Differentiated Thyroid Cancer: The Value of Surgery in Combination with Radioiodine Imaging and Therapy - A German Position Paper from Surgery and Nuclear Medicine. *Nuklearmedizin* **2022**, doi:10.1055/a-1824-1280.	Correction to review not in the field of interest
Hertz, B.; Watabe, T.; Baum, R.P. Celebrating 80 Years Anniversary of Radioiodine for Use in Thyroid Cancer and Perspectives for Theranostics. *Ann Nucl Med* **2022**, *36*, 1007–1009, doi:10.1007/s12149-022-01806-9.	Review not in the field of interest
